# Development of a Communication Protocol for Telephone Disclosure of Genetic Test Results for Cancer Predisposition

**DOI:** 10.2196/resprot.3337

**Published:** 2014-10-29

**Authors:** Linda J Patrick-Miller, Brian L Egleston, Dominique Fetzer, Andrea Forman, Lisa Bealin, Christina Rybak, Candace Peterson, Melanie Corbman, Julio Albarracin, Evelyn Stevens, Mary B Daly, Angela R Bradbury

**Affiliations:** ^1^Department of Medicine, Division of Hematology-OncologyCenter for Clinical Cancer Genetics and Global HealthUniversity of ChicagoChicago, ILUnited States; ^2^Biostatistics FacilityFox Chase Cancer CenterPhiladelphia, PAUnited States; ^3^Department of MedicineDivision of Hematology-OncologyUniversity of PennsylvaniaPhiladelphia, PAUnited States; ^4^Department of Clinical GeneticsFox Chase Cancer CenterPhiladelphia, PAUnited States; ^5^Department of Medicine, Division of Hematology-OncologyDepartment of Medical Ethics and Health PolicyUniversity of PennsylvaniaPhiladelphia, PAUnited States

**Keywords:** genetic testing, test result disclosure, communication, telemedicine, cancer risk assessment, self-regulation theory of health behavior

## Abstract

**Background:**

Dissemination of genetic testing for disease susceptibility, one application of “personalized medicine”, holds the potential to empower patients and providers through informed risk reduction and prevention recommendations. Genetic testing has become a standard practice in cancer prevention for high-risk populations. Heightened consumer awareness of “cancer genes” and genes for other diseases (eg, cardiovascular and Alzheimer’s disease), as well as the burgeoning availability of increasingly complex genomic tests (ie, multi-gene, whole-exome and -genome sequencing), has escalated interest in and demand for genetic risk assessment and the specialists who provide it. Increasing demand is expected to surpass access to genetic specialists. Thus, there is urgent need to develop effective and efficient models of delivery of genetic information that comparably balance the risks and benefits to the current standard of in-person communication.

**Objective:**

The aim of this pilot study was to develop and evaluate a theoretically grounded and rigorously developed protocol for telephone communication of BRCA1/2 (breast cancer) test results that might be generalizable to genetic testing for other hereditary cancer and noncancer syndromes.

**Methods:**

Stakeholder data, health communication literature, and our theoretical model grounded in Self-Regulation Theory of Health Behavior were used to develop a telephone communication protocol for the communication of BRCA1/2 genetic test results. Framework analysis of selected audiotapes of disclosure sessions and stakeholders’ feedback were utilized to evaluate the efficacy and inform refinements to this protocol.

**Results:**

Stakeholder feedback (n=86) and audiotapes (38%, 33/86) of telephone disclosures revealed perceived disadvantages and challenges including environmental factors (eg, non-private environment), patient-related factors (eg, low health literacy), testing-related factors (eg, additional testing needed), and communication factors (eg, no visual cues). Resulting modifications to the communication protocol for BRCA1/2 test results included clarified patient instructions, scheduled appointments, refined visual aids, expanded disclosure checklist items, and enhanced provider training.

**Conclusions:**

Analyses of stakeholders’ experiences and audiotapes of telephone disclosure of BRCA1/2 test results informed revisions to communication strategies and a protocol to enhance patient outcomes when utilizing telephone to disclose genetic test results.

## Introduction

Dissemination of genetic testing for disease susceptibility, one application of “personalized medicine”, holds the potential to empower patients and providers with informed risk reduction and prevention recommendations [1,2]. Genetic testing has become a standard practice in cancer prevention, where genetic testing for cancer susceptibility has become routine for high-risk populations, particularly for breast, ovarian, and colon cancer [2-8]. Heightened consumer awareness of “cancer genes” and other disease susceptibility genomic testing (eg, cardiovascular genetics [9], genetic testing for Alzheimer’s disease [10], and multi-gene genetic testing for cancer and common diseases [11-14]) has escalated interest in and demand for genetic risk assessment. Increasing demand for predictive genetic testing to inform prevention and medical management of cancer and other diseases is expected to surpass accessibility to genetic specialists [15-17].Thus, the promise of personalized medicine will require innovative delivery models for effective, efficient predictive genetic testing and risk communication.

Given the complexity and limitations of genetic testing for disease susceptibility, pre- and post-test counseling are recommended across a variety of fields (eg, cancer, cardiology, and neurology) to optimize patients’ informed consent, understanding of, and adaptive behavioral and psychosocial responses to genetic test results [2,6,11,12,18,19]. Given the complexity of genetic information, the potential for false reassurance, and the potential for psychological distress (eg, persistent anxiety and guilt about the development of cancer in themselves or offspring), communication of genetic test results for cancer susceptibility has traditionally been conducted in-person by health professionals with genetics training [5,20-23]. With increasing demand for genetic testing and time constraints regarding cancer treatment decisions dependent on test results, some genetic counselors are beginning to incorporate telephone disclosure of genetic test results for select patients [24-29]. Some direct-to-consumer companies have incorporated “streamlining” of pre- and post-test counseling protocols [30], offering BRCA1/2 testing, including pre- and post-test counseling entirely by telephone and the Internet. These services are currently commercially available (eg, InformedDNA, Genetic Counseling Services), and some health insurers (eg, Aetna) have partnered with them in the delivery of these services [31-33]. Thus, modifications to traditional genetic service delivery have begun, in the presence of limited data regarding the impact of these changes on patients, providers, and health care systems [31,34]. These changes represent a critical knowledge gap in the translation, implementation, and dissemination of genetic knowledge into effective clinical practice. Concurrently, there is also increasing use of more complex testing, including multi-gene panels evaluating a number of cancer susceptibility loci of varied penetrance, cancer spectrum, and clinical utility [35]. Whole-exome and -genome sequencing add additional complexity with the potential to unveil disease susceptibility beyond the condition of interest [5]. These advances hold great promise to expand the benefits of testing, but in many cases are also associated with greater uncertainty and complexity, presenting additional challenges for providers delivering pre- and post-test counseling [35]. The capacity for traditional counseling models to accommodate these changes is limited. Thus, there is urgent need for theoretically driven studies that evaluate innovations in communication and delivery of cancer genomic advances in real-world clinical settings, which balance the risks and benefits associated with alternatives to in-person communication, in the context of emerging and increasingly complex genomic testing that will address the potential limitations of and inform the adaptive responses to alternative communicative strategies [16,36-38].

We utilized our preliminary data from patients and providers [25], our team’s multidisciplinary expertise, and existing literature of genetic counseling, telephone communication in medical consultations, health communication, and health behavior to develop a communication protocol for telephone disclosure of genetic test results [17,21,39-43]. The goal of this pilot study was to develop and evaluate a protocol for telephone communication of clinical BRCA1/2 (breast cancer) genetic test results that might be broadly generalizable. We utilized our theoretical model to inform short-term cognitive (knowledge) and psychological (state anxiety, general anxiety, and depression) outcomes and to identify audiotaped communication sessions for review. Audiotape reviews and direct stakeholder (patient and provider) feedback were utilized to evaluate and inform refinements to our initial telephone communication protocol [34].

## Methods

### Theoretical Model

**Figure 1:**
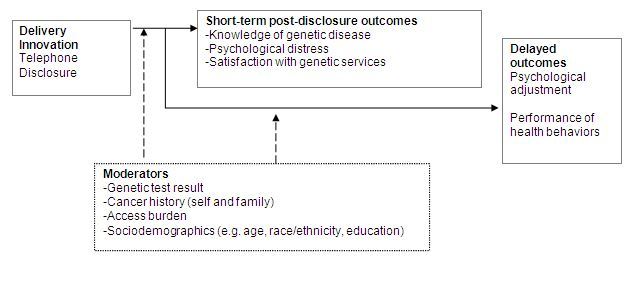
Theoretical model to evaluate innovations to delivery of genetic information (guided by the Self-Regulation Model of Health Behavior).

This study and related work [25,34,44,45] is informed by our theoretical model to optimize and evaluate the outcomes of innovation to delivery of genetic services (Figure 1). The health benefits of genetic testing for disease susceptibility are expected to be greatest when there are successful interventions to modify disease susceptibility and thus, to improve patient outcomes [11,46,47]. As improved patient outcomes are contingent upon high-risk individuals adopting preventive or promotive health behaviors, the effectiveness of genetic testing for disease susceptibility is contingent upon successful behavior modification. Our model is grounded in the Self-Regulation Theory of Health Behavior (SRTHB), which has been utilized in descriptive and intervention-based research involving the study of health threats and health behavior [39,48-51]. Simplistically, this theory proposes that the performance of a health behavior is the product of an individual’s knowledge and perceptions of the disease threat (eg, genetic risk of disease) and the health behavior (eg, risk reduction behaviors) and the biopsychosocial impact of the health behavior [39,40,49,51-53]. Importantly, the SRTHB emphasizes common-sense representations rather than medical or scientific definitions, and incorporates individual biological, cognitive, emotional, familial, and cultural experiences that might contribute to individual variability in knowledge and perceptions of genetic information, disease etiology, and controllability, and the impact of knowledge and perceptions on risk modification for individuals and their families. It has been proposed that the SRTHB is an ideal framework for considering the psychosocial and behavioral outcomes and thus, the effectiveness of genetic screening for disease susceptibility in the era of personalized medicine [40,42,43]. The SRTHB and associated literature suggests that the performance of risk reduction behaviors and psychosocial adjustment to communication of genetic test results is an iterative process, in which proximal perceptions of and responses to risk information shape more distal behavioral and psychosocial outcomes [39,42,43,54,55]. Thus, the SRTHB suggests an innovative model for the evaluation of both the short-term and long-term responses to novel delivery methods of genetic services, communication of genetic test results, and the impact of that communication on health behaviors (Figure 1).

The literature and our preliminary data support the hypothesis that both short-term cognitive (knowledge and perception) and psychological (state anxiety, general anxiety, and depression) outcomes and longitudinal adjustment and performance of surveillance behaviors, in response to receipt of genetic test results, will be moderated by biological (test result [25,56-58], cancer history [57,59-61]), sociodemographic (eg, race/ethnicity [57,61-63], access burden [25,56]), cognitive, and emotional [26,27,64] factors. Thus, while innovations to delivery of genetic test results might provide equal outcomes in a broad population, there might be subgroups (ie, moderators) for whom this innovation to communication of genetic information is particularly harmful or particularly useful. If the promise of genomics to improve population health is to be realized broadly, it will be critical to understand those factors that have the potential to moderate that outcome for some, and to incorporate that knowledge into the design of future interventions for delivery of genomic information.

### Participants

Institutional Review Board approval was obtained before initiating this study. Participants were recruited through the Fox Chase Cancer Center Risk Assessment Program between September 2009 and July 2010. Eligible patients were over 18 years old, could communicate in English, completed pre-test counseling with a genetic counselor, and elected to proceed with BRCA1/2 testing. Consistent with professional guidelines from the National Society of Genetic Counselors and clinical services at Fox Chase Cancer Center, key components of in-person pre-test counseling included ascertainment of targeted medical and family history, assessment of cancer risk, education on cancer genetics, discussion of appropriate genetic testing options, and informed consent for genetic testing [19,65]. Eligible patients were approached by research staff at completion of pre-test counseling offering the opportunity to participate in a study receiving their BRCA1/2 test results by telephone and returning for in-person medical management recommendations. Written informed consent from patients was obtained prior to conducting audiotaped telephone disclosure sessions. Providers also signed informed consent and completed brief surveys assessing their perception of how participants perceived their disclosure sessions.

### Telephone Disclosure Communication Protocol

We utilized our preliminary data from patients and providers [25], our team’s multidisciplinary expertise, existing literature of genetic counseling, health communication, telephone communication in medical consultation, and our theoretical model grounded in SRTHB to develop a protocol for telephone disclosure of genetic test results [5,17,21,39-43,66,67]. The key components, although tailored for this study to the disclosure of BRCA1/2 testing results by telephone, were designed to be broadly adaptable to communication of genetic/genomic test results, hereditary risk, and risk reduction strategies for other heritable diseases. Key components include (Table 1): “Visual Aids”, “Standardized Communication Topics”, and “Provider Probes”; “standard probes” are used intermittently throughout the session to assess patient understanding (eg, “What questions do you have for me before we go on?”) and affect (eg, “How are you feeling now that you know…?”). At the conclusion of the session, a “Teach Back” probe (eg, “Please tell me in your own words your understanding of your genetic test results and what those results mean for you”) is used to assess comprehension [67]; “situational probes” are used as needed (eg, “It sounds like this might not be the best time for us to talk. Is there another time that would work better for you?”) in contexts where the situation might present challenges to optimal outcomes. Other key components include “Provider Training”: in-person training was provided to genetic counselor (GC) providers to optimize the translation and implementation from in-person to telephone disclosure; and “Quality Assurance and Protocol” evaluation where all telephone disclosure sessions were audiotaped. Sessions’ meeting criteria (see Statistical Analyses) were reviewed by research staff in tandem with disclosure checklists completed by the genetic counselors to (1) ensure inclusion of 12 key components of disclosure [34], and (2) inform potential refinements to the communication protocol. Additionally, all participants who completed telephone disclosure were asked to return for an in-person follow-up appointment with a physician to address any remaining questions and to discuss and implement medical recommendations for cancer screening and/or risk reduction strategies (ie, prophylactic surgery and/or chemoprevention) [34].

**Table 1:** Key components of telephone communication protocol for BRCA1/2 testing.

Components		
**Visual Aids**		
		Pedigree
		Etiology
		Heritability
		Associated cancer risks
		Sample genetic test results
		Risk reduction options
**Standardized Communication Topics**		
		Confirm patient’s identity
		Introduce all participants
		Assess adequacy of hearing and access to Visual Aids
		Affirm session purpose and patient’s desire for results
		Provide test results, interpretation, and implications
**Provider Probes**		
	**Standard - Evaluate patient:**	
		Understanding
		Emotional response
	**Situational - Address:**	
		Session distractions
		Patient interruptive
		Patient emotional
		Patient disengaged
		Others present in session reactions/needs
	**Teach Back:**	
		Evaluate patient understanding at conclusion
**Provider Training**		
	**In-person**	
		Training manual
		Challenges to telephone communication
		Mock telephone disclosure w/Individualized feedback
**Quality Assurance**		
	**Evaluation**	
		Innovations in delivery of genetic/genomic information
		Inclusion of key components
**In-Person Follow-up**		
	**Appointment with physician**	
		Address remaining questions
		Discuss and implement medical management recommendations

### Measures

#### Overview

Patients completed quantitative assessments of knowledge [68-70], psychological distress (state anxiety) [71], psychological adjustment (general anxiety and depression) [72], and satisfaction with genetic services [73-75] previously reported [44] within 3-5 days after their pre-test counseling session (baseline) and telephone disclosure. Participants completed 7 selected items consistent with the National Institute of Health National Center for Human Genome Research Cancer Genetics Consortium Knowledge scale and utilized in prior research [69], including items evaluating mechanism of cancer inheritance (1 item), and the meaning of positive (3 items) and negative (3 items) results. Internal consistency in this study was good (mean alpha=.72).

#### Psychological Distress

State anxiety was measured with the 20-item State Inventory of the State-Trait Anxiety Inventory (STAI), which is a sensitive indicator of transient or situational changes in anxiety (test-retest reliability: *r*=16 - .62) experienced by patients in response to stressful procedures or life events [71] and is frequently used to assess the short-term response to the receipt of a genetic test result [57,76]. Internal consistency in this study was high (mean alpha=.95). General anxiety and depression were assessed with the 7-item Hospital Anxiety and Depression Scale (HADS) anxiety and depression subscales, which have been utilized in the general population and a wide range of medical patients, including those with cancer [72,77]. Internal consistency in this study was good for both the anxiety and depression subscales (mean alpha=.88, .78, respectively) [34].

#### Satisfaction With Genetic Services

Satisfaction with health communication was measured with a 9-item scale, reflective of constructs identified in prior qualitative, quantitative, and comparative research evaluating participants’ perceptions of their genetic counseling and testing experience, including cognitive, affective, and time/attention items [73-75]. Internal consistency in this study was good (mean alpha=.73) [34].

#### Opinions and Experiences Regarding Telephone Disclosure Post-Disclosure Only

Patients and providers also completed parallel open-ended questions after telephone disclosure to elicit patient experiences (3 items), GC experiences (3 items), and GC perceptions of patient experiences (3 items) with, and suggestions for improving their telephone disclosure session [34].

### Statistical Analyses

Changes in pre-test and 3-5 days post telephone disclosure scores were calculated for each participant and each construct, which are reported separately [34]. Pre-defined criteria were developed to select telephone disclosure sessions for review to inform modifications to the communication protocol [34]. These included (1) all positive results (n=9), (2) discordance between patient reported satisfaction, perceived understanding/ emotional response, and provider perceptions of patient satisfaction/ understanding/ emotional responses; discordance was defined as a difference of >2 points between patient and provider for any individual item (n=12), (3) provider request (n=1), (4) decline in knowledge in the lowest 10 percentile; these included a decline in knowledge of >3.3 points (n=8), and (5) increase in state anxiety, general anxiety, or depression in highest 10 percentile (n=21). These included increases in state anxiety scores >11.3 points, HADS-anxiety, or HADS-depression subscale scores >3 points. These selection criteria resulted in review of 38% (33/86) of recorded telephone disclosures. Participants selected for review did not differ statistically from those not selected for review on race, age, education, test result, cancer history, treatment decision, and known familial mutation.

Framework analysis was utilized to analyze open-ended responses regarding patient and GC reported advantages, disadvantages, and recommended modifications (eg, “What did you/your patient like/dislike about receiving genetic test results by telephone? What changes would you recommend?”) [36,57,78-80]. The intent of this analysis was to identify themes that might not have been represented in quantitative surveys, informing modifications to the intervention from both patient and provider perspectives. Two investigators, a clinical health psychologist trained in health outcomes research, and a medical oncologist, each with extensive clinical experience in the delivery of hereditary cancer risk information, independently reviewed responses utilizing thematic analysis to record primary and secondary themes for each open-ended item.

## Results

### Participant Characteristics

A total of 167 eligible subjects were approached for participation. Of the 167 eligible subjects, 100 (59.9%) agreed to participate, provided informed consent, and completed the baseline assessment, and 95 proceeded with BRCA1/2 testing [34]. A total of 86 participants completed both baseline and post-telephone disclosure surveys, and participant characteristics are described in Table 2.

All participants were women, 10% (9/86) were non-white, 59% (51/86) had a personal history of cancer, and 50% (43/86) had a college degree or graduate education. The majority of participants (83%, 71/86) received an uninformative negative result (ie, negative BRCA1/2 result with no known clinically significant mutation in the family). Nine women received a positive result (ie, clinically significant BRCA1/2 mutation), 4 received a true negative result (ie, negative for a known clinically significant mutation in the family), and 2 received a variant of uncertain significance. Provider checklists revealed high fidelity to communication topics [34].

**Table 2:** Participant characteristics (participants who completed both pre and post disclosure assessments) (n=86).

Characteristic		n (%)
Age, median (range)		49 (24-73)
**Race**		
	White	77 (90)
	Black / African American	6 (7)
	Asian	3 (3)
**Education**		
	High school only	13 (15)
	Some college / vocational	30 (35)
	College degree	21 (24)
	Graduate degree	22 (26)
Marital status: married/domestic partnership		54 (62)
History of cancer		51 (59)
Treatment decision^a^		19 (22)
Known mutation in family		6 (7)
**Genetic test (BRCA 1/2) result**		
	Indeterminate (uninformative negative)	71 (83)
	Positive	9 (10)
	True negative	4 (5)
	Variant of unknown significance	2 (2)

^a^Defined as individuals who had not received definitive surgical treatment for their breast cancer at the time of initial counseling.

### Patient and Provider Open-Ended Responses

Patients and providers identified several advantages for patients to telephone disclosure of genetic test results, including patient conveniences, setting, and timing. Patient and provider reported advantages for patients did not differ. Providers also identified advantages of telephone disclosure for genetic counselors, including setting, scheduling, and efficiency (Table 3). Most patients and genetic counselors reported no disadvantages to telephone receipt of test results for patients. Primary themes reported by patients and genetic counselors were communication, delivery, and patient specific challenges (Table 3). Providers reported similar disadvantages for genetic counselors, which also focused on communication and delivery challenges, as well as patient specific factors, such as providing a positive or variant of uncertain significance (VUS) result.

**Table 3:** Advantages and disadvantages to telephone disclosure: open-ended survey responses from patients and providers.^a^

Advantages / disadvantages	
**Patient advantages reported by patients and genetic counselors/providers**	
	*Patient conveniences*, eg, less travel, lost work time, need for dependent care
	*Setting*, eg, being in a comfortable environment, having a support person available
	*Timing*, eg, receiving results prior to MD appointment
**Provider advantages reported by genetic counselors/providers**	
	*Scheduling*, eg, greater availability and flexibility for scheduling patient appointments
	*Setting*, eg, having access to resources in provider’s office during appointment
	*Efficiency*, eg, no travel to and wait in clinic
**Patient disadvantages reported by patients and genetic counselors/providers**	
	*Communication*, eg, not being able to see the genetic counselor for nonverbal communication
	*Delivery*, eg, interruptions (workplace, children in the home)
	*Patient-specific factors*, eg, receiving positive or uncertain results
**Provider disadvantages reported by genetic counselors/providers**	
	*Communication*, eg, not being able to see the patient for nonverbal communication
	*Delivery*, eg, interruptions (workplace, children in the home)
	*Patient-specific factors*, eg, delivering positive or uncertain results

^a^From 86 encounters (n=86 patients, n=4 providers).

### Modifications to the Communication Protocol

Modifications to the original communication protocol were informed by the audiotape review (33/86, 38%) as described above, open-ended survey responses from patients (n=86) and providers (n=4) on 86 disclosure sessions, our theoretical model, and the literature, and developed by a clinical health psychologist. Primary modifications are summarized in Table 4 and included GC clarification of patient instructions for telephone disclosure during pre-test counseling (eg, reviewing visual aids), and appointments for telephone disclosure scheduled to allow patients to plan to be in a private, comfortable place where they would be free of interruptions. Scheduling the disclosure session also allowed patients to include significant others in the session. Additionally, it had the potential to reduce anticipatory anxiety and eliminate the frustration of “playing phone tag”. Other modifications included refinement of visual aids, including more clearly labeling mock test report forms to avoid patient confusion, and more detailed telephone disclosure checklists were created for GCs to ensure the communication protocol’s coverage of all critical elements of disclosure (eg, Genetic Information Nondiscrimination Act, or GINA), to enhance fidelity to the protocol (eg, reminder that session will be audiotaped), and to facilitate use of verbal knowledge and affective probes, critical to the assessment of understanding and emotional response in the context of telephone communication where nonverbal cues are not available for this purpose. Another modification included GC training to facilitate identification and management of situations that might present unique challenges in the context of telephone communication. Situations addressed included identifying (1) patients’ biopsychosocial risk factors prior to disclosure session (eg, personal/familial cancer history; baseline high anxiety or low health literacy; uninformative test results, or need for additional testing), (2) signs of potentially poorer affective and cognitive responses during result disclosure (eg, silence, pressured speech, incongruous affect), and (3) need for session management (eg, interruptions), in the absence of the visual information providers are accustomed to incorporating into their assessments of patients in the conduct of in-person sessions. Training included, in addition to regular utilization of affective and knowledge probes and the “teach-back”, increased attention and response to auditory cues in patients’ speech, eg, rate, pitch, volume, amount, type and congruity; and utilization of situational probes, eg, “Did I catch you at an inconvenient time…”, “Would it be ok to check in now with your (spouse, mother, sister, etc?)”, “Is this the information you were expecting, or how can I better help you meet your needs?”

**Table 4:** Modifications to communication protocol resulting from stakeholder (patients and genetic counselors) surveys (n=86) and session tape reviews (n=33).^a^

Modifications to the communication protocol	Reason for tape review	Reviewer observations	Patient comments	GC^b^comments
1. Clarified telephone disclosure (TD) instructions in pre-test counseling			-Visual aid: read in advance and have for session	-Visual aid: read in advance and have for session
			-Schedule sufficient time for session and processing	-Schedule sufficient time for session and processing
			-Have support person	-Have support person
2. Scheduled TD appointments	-Increase in anxiety or depression or decline in knowledge	Session occurred in non-private environment without visual aids	-Session was disrupted (eg, workplace, childcare)	-Session was disrupted (eg, workplace, childcare)
			-Session disrupted other activities	-Difficult to reach patient (“phone tag”)
3. Refined visual aids			Visual aids confusing	Visual aids confusing
4. Improved disclosure checklist:	-Increase in anxiety	-Some elements of disclosure checklist omitted		Who else is on call/present?
-Enhanced formatting				
-Included information on GINA^c^		-Patient concerned about genetic discrimination		
5a. GC training:	-Increase in anxiety or depression	-High baseline anxiety	-Communication challenging without visual cues	Interpreting patient affective response and providing emotional support challenging without visual cues
-Recognizing signs of negative affect in the absence of visual cues	-Patient/GC discordance	-Inaccurate expectations		
-Effective use of affective and situational probes				
-Need to pace session to meet patient needs				
-Identifying risk factors for negative affective response	-Positive test result	-Personal history of cancer	-Need to be prepared for all results	
		-Treatment decision pending		
		-Family history (uninformative or extensive cancer)		
		-Need for additional tests (self or family members)		
5b. GC training:	-Decline in knowledge	Low health literacy		Interpreting patient cognitive response and providing remediation challenging without visual cues
-Identifying risk factors for confusion				
-Recognizing signs of confusion				
-Techniques to improve patient comprehension	-VUS^d^test result			
-Effective use of knowledge and situational probes and “teach back” to assess understanding				
5c. GC training:	-Decline in knowledge			-Responding to challenging patients/situations
-Effective use of situational probes to control situation				-Controlling the session
6. Conduct larger trial to evaluate outcomes for potentially vulnerable subgroups	-Positive test result	-Need for additional testing (self or family members)	TD might be more challenging in some situations (eg, Positive test result, need for additional testing, poor understanding after pre-test counseling, psychological factors)	TD might be more challenging in some situations (eg, Psychosocial comorbidities, English as a second language, pending treatment decisions, personal cancer history)
	-VUS test result	-Personal history of cancer		
		-Family history of cancer		
	-Increase in anxiety or depression	-Uninformative negative result		
		-Insurance issues		

^a^Based on a priori review criteria

^b^GC: genetic counselor

^c^GINA: Genetic Information Nondiscrimination Act

^d^VUS: variant of uncertain significance

## Discussion

### Principal Findings

The goal of this pilot study was to evaluate stakeholder (patient and provider) experiences with our telephone disclosure protocol, grounded in our theoretical model, to inform refinements to our initial protocol for the delivery of genetic test results and to obtain preliminary outcome data for future research. To our knowledge, this is the first study to describe the development and refinement of a telephone communication protocol for the disclosure of genetic test results informed by provider and patient feedback, as well as systematic review of communication sessions grounded in a theoretical model of health behavior [42,43]. This study provides a standardized model for optimizing telephone communication that will allow future studies to implement and evaluate the efficacy, risks, and benefits of telephone disclosure of genetic test results. This protocol includes a number of innovations made in response to our findings, supported by our theoretical model and the literature regarding inclusion of telephone communication into primary care. These include the need for specific training for providers to learn compensatory techniques for telephone communication [34,66] (eg, the need to implement more verbal probes in the absence of nonverbal cues) [26,27,34,64,81,82], and have suggested that telephone disclosure might provide equal or improved outcomes when compared to in-person disclosure [26,27,64,83]. This is consistent with our data [34,84] and primary care literature suggesting that telephone might be better suited to encounters following an initial in-person consultation [66]. Given an increased demand for genetic counseling services and the increasing availability of many new modes of genetic and genomic testing (eg, multi-gene, germline, tumor, and pharmacogenomic testing), our study has focused on reconciling provider and patient perspectives on telephone communication to formulate an effective and efficient telephone communication protocol delivery of genetic test results.

The results of our study indicate that patients and providers identify both advantages and disadvantages of telephone disclosure of genetic test results (Table 3). Advantages, highlighted by both patients and providers, included the convenience, scheduling, setting, timing, and efficiency of telephone disclosure. Perceived disadvantages to telephone communication, associated with the loss of nonverbal communication, included the possibility that some subgroups of patients (eg, positive test results, pre-existing psychosocial or cognitive comorbidities) might be more vulnerable to poorer outcomes with telephone communication of genetic test results. In the absence of a randomized trial, it is not possible to know if patients in these subgroups might also have fared differently from others had they received their results in person. Patient and provider feedback and review of disclosure session audiotapes suggested many modifications that are expected to optimize communication of genetic test results by telephone. The results of our study illustrate the challenges in assessing patients’ emotional response and understanding via telephone and providers’ response to those patient needs. They emphasize the importance of identifying and developing the training needed to establish skills necessary for novel modalities of communication of genetic/genomic information.

To address these challenges, provider training, including additional verbal probes and procedures to assess patients’ psychological response and understanding, as well as situations unique to non-in person communication, must be incorporated into counseling to ensure effective communication of health information via telephone. Professional or educational training for alternative counseling models that incorporate these probes and procedures could prove invaluable for future counseling for both novice and experienced genetic counselors. Evolving genetic technologies have the potential to expand the reach of genetic testing. Realizing the associated health gains will require a workforce of genetic counselors with training in telephone and other nontraditional models of communication of genetic information. Research has shown that effective communication between providers and patients can be achieved through these technologies and might result in improved outcomes [85]. Future studies must focus on evaluating the potential disadvantages of telephone disclosure for vulnerable subgroups in randomized clinical trials.

In this paper, we present a model for adapting the standard genetic counseling model for innovations in the delivery of genetic medicine. Our model includes stakeholder (patient and provider) input, as well as systematic review of audiotapes of pilot sessions of telephone disclosure of genetic testing results and is theoretically informed. We expect this model will be broadly applicable to adaptions of counseling for other innovations in the delivery of genetic/genomic test results. For example, we are currently employing adaptations of this model to study the potential to increase access by utilizing telemedicine to deliver genetic counseling services to community practices where genetic counselors are not available [45], to inform modifications to genetic counseling and delivery of multi-gene testing for cancer susceptibility for patients with suspected hereditary breast and ovarian cancer (HBOC) and other hereditary cancer syndromes, including hematological and gastrointestinal syndromes, and to inform prescriptive decisions for providers. We expect this model to be broadly generalizable to evolving contexts in genetics and medicine as innovations develop.

### Limitations

We acknowledge several limitations to this study. Despite the recruitment of a clinical population, we had relatively low representations of some subgroups (eg, ethnic minorities and those of low socioeconomic status). Thus, our findings may not be generalizable to a more diverse population. Our on-going multi-center randomized study aims to increase representation of these subgroups. It will also inform how cognitive and affective responses might differ between patients receiving in-person and telephone communication of genetic test results.

### Conclusions

Providers delivering and patients receiving genetic test results by telephone identified advantages to telephone disclosure (eg, perceived convenience for patients and providers) and disadvantages (eg, loss of non-verbal cues to assess and communicate emotional responses and understanding). Review of stakeholder reported experiences and audiotapes of telephone disclosures lead to a number of communication strategy and protocol revisions to enhance patients’ cognitive, affective, and behavioral responses to genetic test results in the context of telephone disclosure of genetic test results.

## Abbreviations

GC: genetic counselor
GINA: Genetic Information Nondiscrimination Act
HADS: Hospital Anxiety Depression Scale
SRTHS: Self-Regulation Theory of Health Behavior
STAI: State-Trait Anxiety Inventory
TD: telephone disclosure
VUS: variant of uncertain significance
